# Accomplishing Career Transitions 2019: facilitating success towards the professoriate

**DOI:** 10.1186/s12919-021-00220-9

**Published:** 2021-06-22

**Authors:** Verónica A. Segarra, Jim Vigoreaux, Maria Elena Zavala, Ashanti Edwards

**Affiliations:** 1 American Society for Cell Biology, Bethesda, MD 20814 USA; 2 Department of Biology, High Point University, High Point, NC 27268 USA; 3 Department of Biology and Office of the Provost, University of Vermont, Burlington, VT 05405 USA; 4 Department of Biology, California State University Northridge, Northridge, CA 91330 USA

**Keywords:** Professional development for scientists, Tenure-track faculty, Tenure, Professoriate, Professional development practicum, Professional development experiential learning, Accomplishing Career Transitions, American Society for Cell Biology, Minorities Affairs Committee

## Abstract

The Minorities Affairs Committee of the American Society for Cell Biology through its Accomplishing Career Transitions (ACT) program aims to ease critical transitions for postdocs and junior faculty from underrepresented backgrounds in STEM or from minority-serving institutions as they work towards promotion and tenure at a wide range of academic institutions. The ACT program is a 2-year cohort-based professional and skills development program that kicks off with a summer workshop and continues with additional online training sessions on selected topics, forging the creation of a permanent mentoring community for the participants. In this BMC Proceedings Supplement, we highlight selected content from the first ACT summer workshop held in 2019 at the Rizzo Center in Chapel Hill, NC. The goal of this BMC Proceedings Supplement is to amplify impact of ACT programming in a way that transcends the ACT Fellow community to benefit an increased number of scientists.

## Background

The Minorities Affairs Committee (MAC) of the American Society for Cell Biology (ASCB) through its Accomplishing Career Transitions (ACT; https://www.ascb.org/career-development/2021-accomplishing-career-transitions-act-program/) program aims to ease critical transitions for postdocs and junior faculty from underrepresented backgrounds in STEM or from minority-serving institutions (MSIs) as they work towards promotion and tenure at a wide range of academic institutions. Scientists from underrepresented backgrounds in STEM or from MSIs tend to have less access to mentoring than their peers from well-represented groups or their institutions are under-resourced and cannot provide relevant mentoring and professional development activities, respectively [1, 2]. The ASCB MAC has a long history in creating professional development programs to help relieve these disparities in access to mentoring and professional development resources [3–6].

The ACT program is a 2-year cohort-based professional and skills development program that kicks off with a summer workshop and continues with additional online training sessions on selected topics, forging the creation of a permanent mentoring community for the participants. ACT combines five key elements into the framework of what has worked best in past ASCB MAC professional development workshops: 1) Core sessions to help all trainees develop transferrable entrepreneurial skills such as leadership, communication, and negotiation; 2) Modular parallel sessions that allow participants to select the most appropriate session based on their academic career path (e.g. teaching-intensive or research-intensive) and career stage (e.g. postdoc or junior faculty); 3) Customized content in core and parallel sessions to meet the needs and challenges specifically articulated by participant trainees immediately before the summer workshop; 4) Opportunity for skill application through a practicum intended to benefit participating trainees; and 5) Scaffolded expansion of trainees’ professional networks, including peer- and near-peer mentors. In this BMC Proceedings Supplement, we highlight selected content from the first ACT summer workshop held in 2019 at the Rizzo Center in Chapel Hill, NC. The goal of this BMC Proceedings Supplement is to amplify impact of ACT programming in a way that transcends the ACT Fellow community to benefit an increased number of scientists.

The topics encompassed in this Supplement are relevant now in 2021 more than ever, as trainees adapt to the effects of the COVID-19 global pandemic crisis in their career trajectories. Topics include effective mentorship, obtaining a faculty position, starting a lab, preparing for tenure and promotion, and professional development through experiential learning. These topics are discussed in the context of both teaching-intensive and research-intensive academic aspirations. The authors have grounded their discussion of these topics in relevant literature and resources. Our purpose is to empower trainees with tools to understand success in these areas in the context of their institutions, not as prescriptive, but as unique experiences that they can tune to resonate with their values, personal trajectories and definitions of success.

## Data Availability

Not applicable.

## Abbreviations

ACT: Accomplishing Career Transitions
ASCB: American Society for Cell Biology
MAC: Minorities Affairs Committee.
